# Supplementary material for: EZH2/H3K27Me3 and phosphorylated EZH2 predict chemotherapy response and prognosis in ovarian cancer
Source: PeerJ. 2020 May 12;8:e9052. doi: 10.7717/peerj.9052 (PMC7227641; doi:10.7717/peerj.9052)

IHC of EZH2

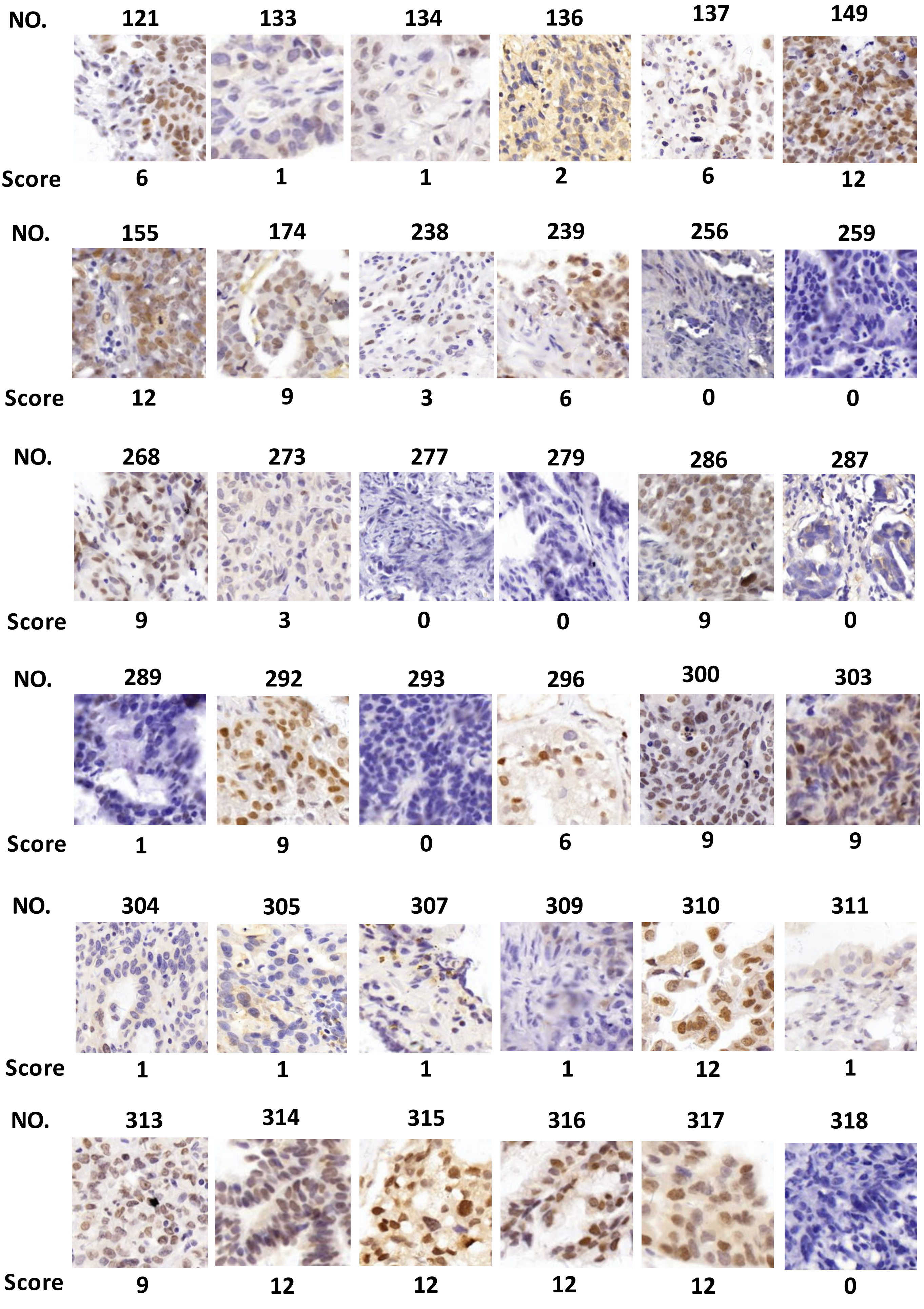
NO.
121
133
134
136
137
149
Score
6
1
1
2
6
12
NO.
155
174
238
239
256
259
Score
12
9
3
6
0
0
NO.
268
273
277
279
286
287
Score
9
3
0
0
9
0
NO.
289
292
293
296
300
303
Score
1
9
0
6
9
9
NO.
304
305
307
309
310
311
Score
1
1
1
1
12
1
NO.
313
314
315
316
317
318
Score
9
12
12
12
12
0

IHC of EZH2

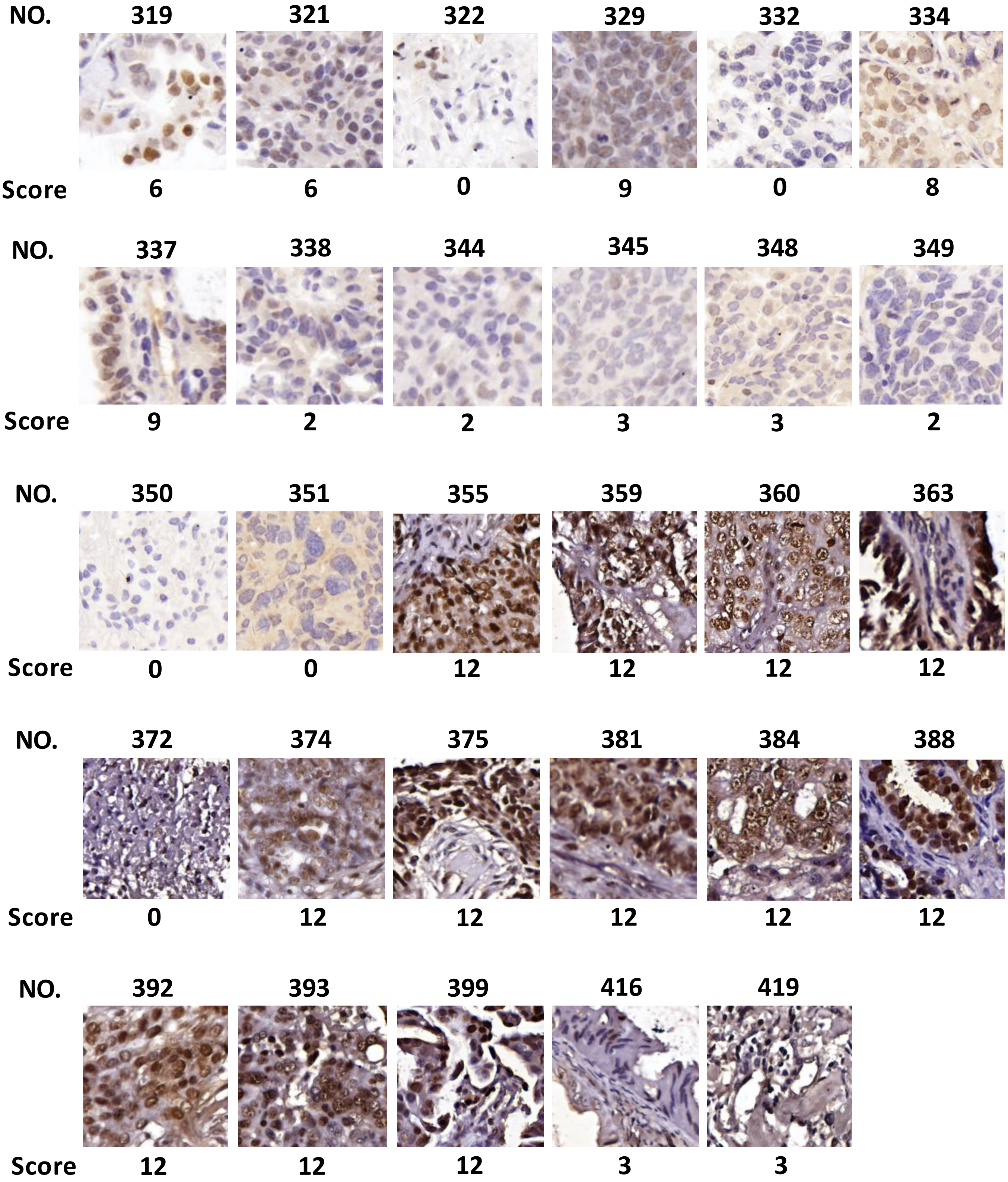
NO. 319 321 322 329 332 334
Score 6 6 0 9 0 8
NO. 337 338 344 345 348 349
Score 9 2 2 3 3 2
NO. 350 351 355 359 360 363
Score 0 0 12 12 12 12
NO. 372 374 375 381 384 388
Score 0 12 12 12 12 12
NO. 392 393 399 416 419
Score 12 12 12 3 3

IHC of pEZH2

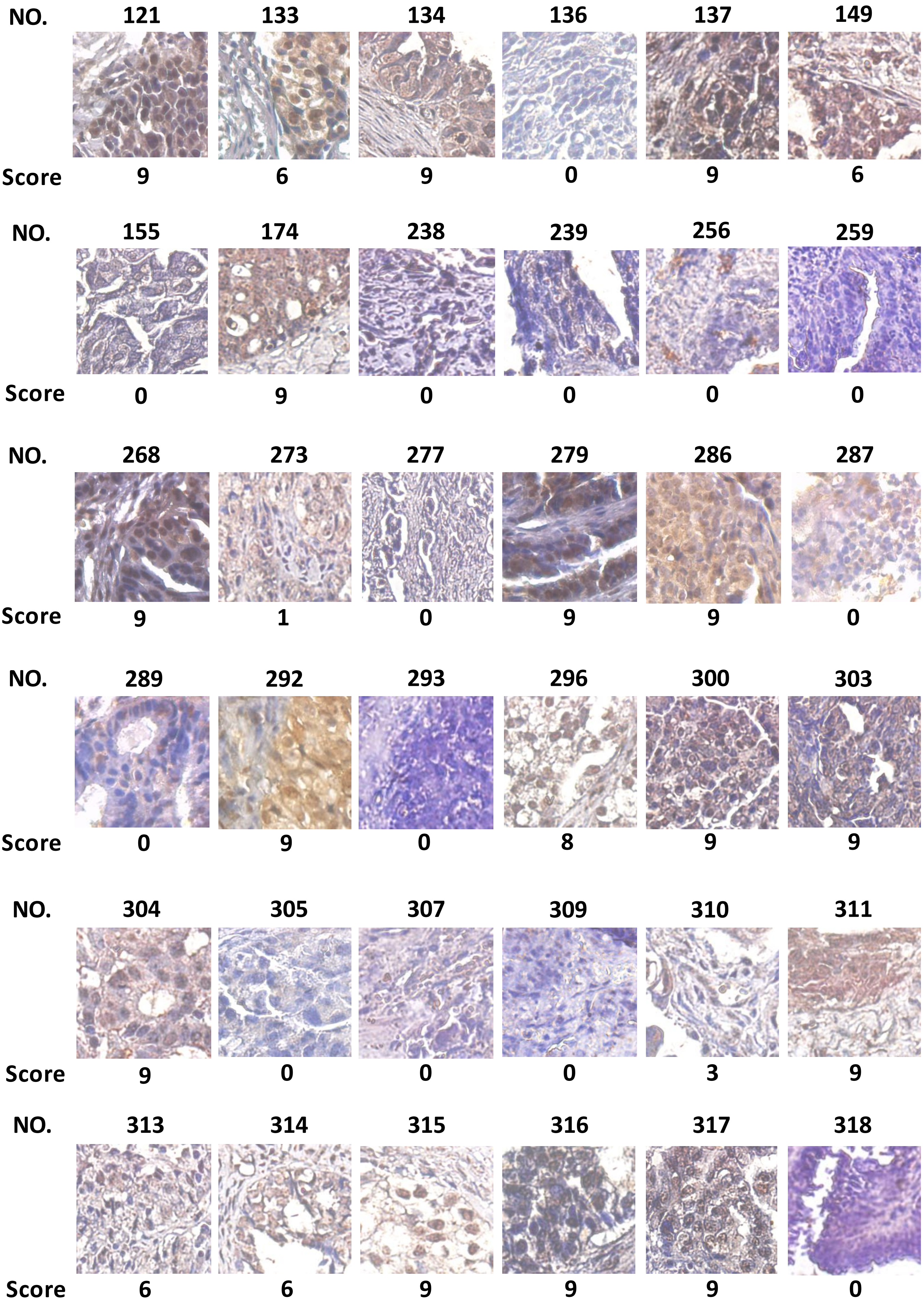
NO. 121 133 134 136 137 149
Score 9 6 9 0 9 6
NO. 155 174 238 239 256 259
Score 0 9 0 0 0 0
NO. 268 273 277 279 286 287
Score 9 1 0 9 9 0
NO. 289 292 293 296 300 303
Score 0 9 0 8 9 9
NO. 304 305 307 309 310 311
Score 9 0 0 0 3 9
NO. 313 314 315 316 317 318
Score 6 6 9 9 9 0

IHC of pEZH2

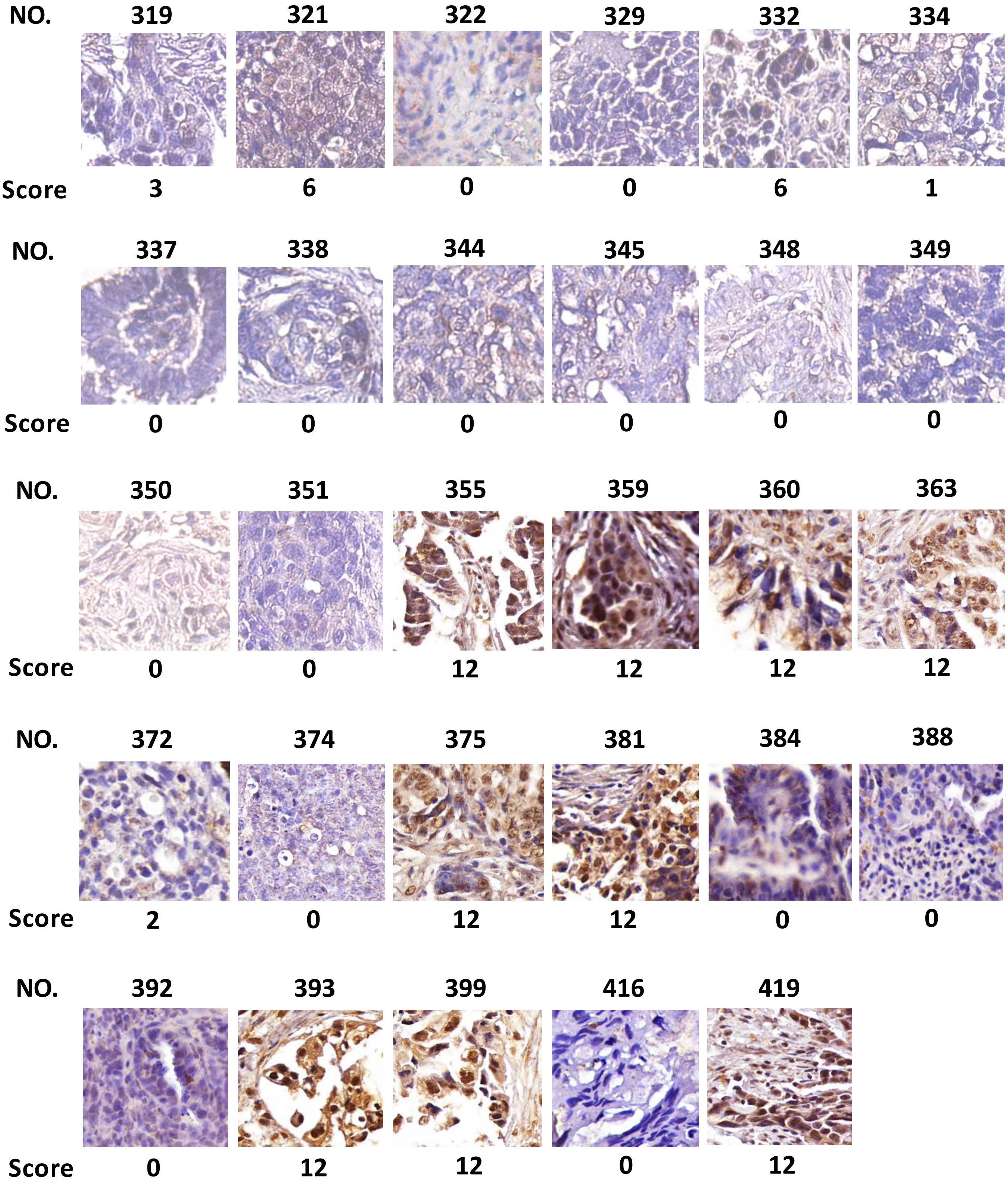
NO. 319 321 322 329 332 334
Score 3 6 0 0 6 1
NO. 337 338 344 345 348 349
Score 0 0 0 0 0 0
NO. 350 351 355 359 360 363
Score 0 0 12 12 12 12
NO. 372 374 375 381 384 388
Score 2 0 12 12 0 0
NO. 392 393 399 416 419
Score 0 12 12 0 12

IHC of pAkt1

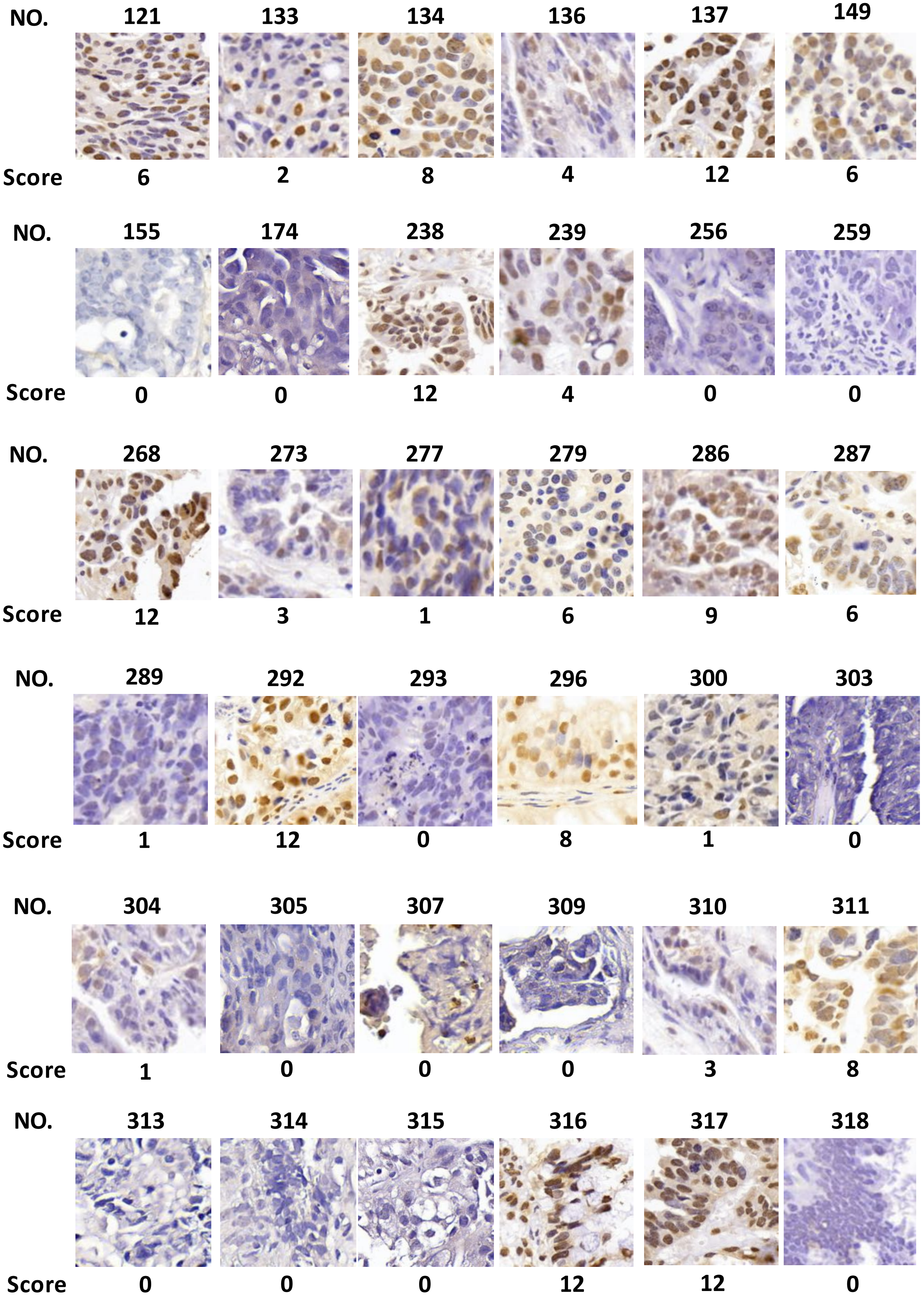
NO. 121 133 134 136 137 149
Score 6 2 8 4 12 6
NO. 155 174 238 239 256 259
Score 0 0 12 4 0 0
NO. 268 273 277 279 286 287
Score 12 3 1 6 9 6
NO. 289 292 293 296 300 303
Score 1 12 0 8 1 0
NO. 304 305 307 309 310 311
Score 1 0 0 0 3 8
NO. 313 314 315 316 317 318
Score 0 0 0 12 12 0

IHC of pAkt1

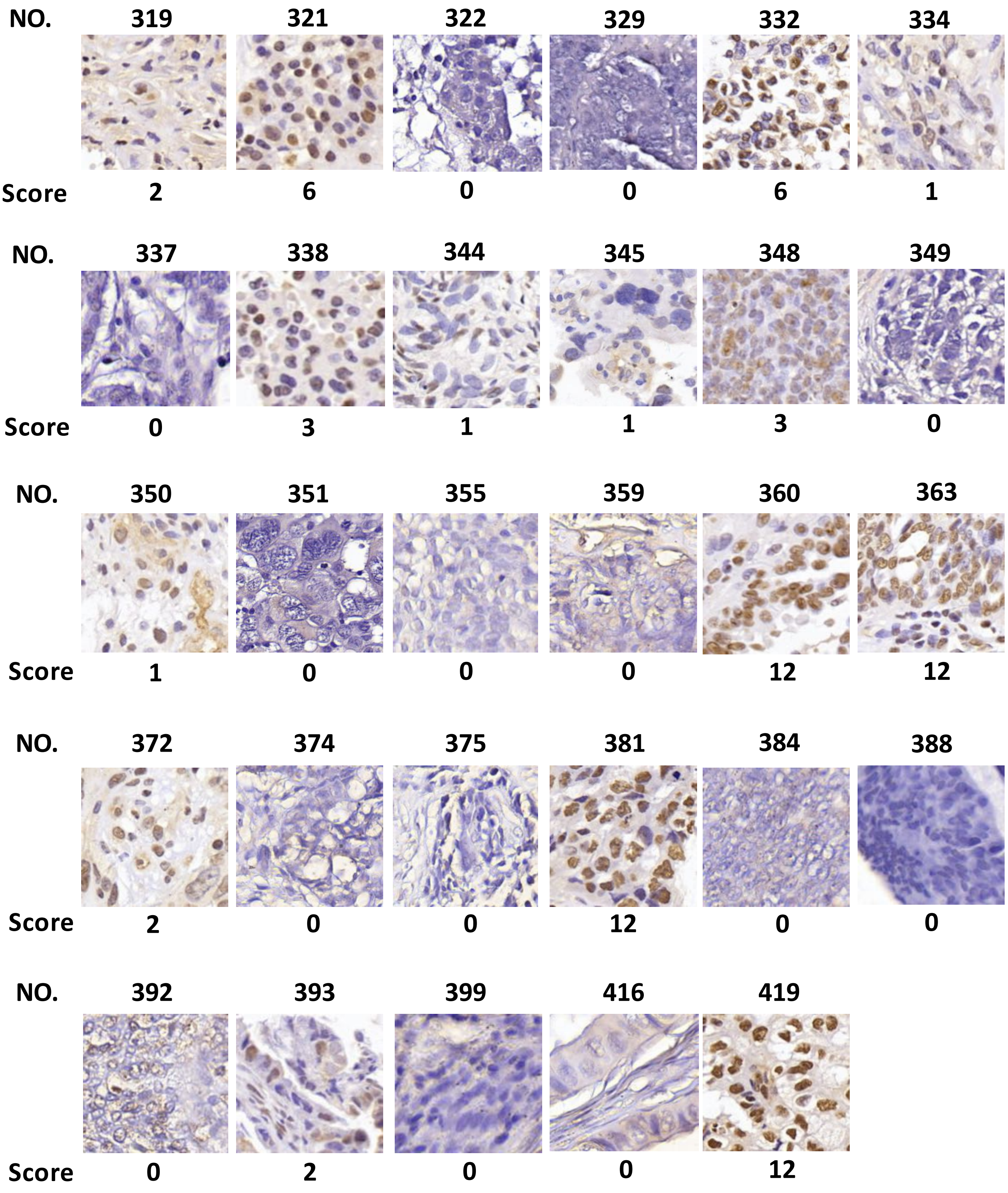
NO. 319 321 322 329 332 334
Score 2 6 0 0 6 1
NO. 337 338 344 345 348 349
Score 0 3 1 1 3 0
NO. 350 351 355 359 360 363
Score 1 0 0 0 12 12
NO. 372 374 375 381 384 388
Score 2 0 0 12 0 0
NO. 392 393 399 416 419
Score 0 2 0 0 12

IHC of H3K27me3

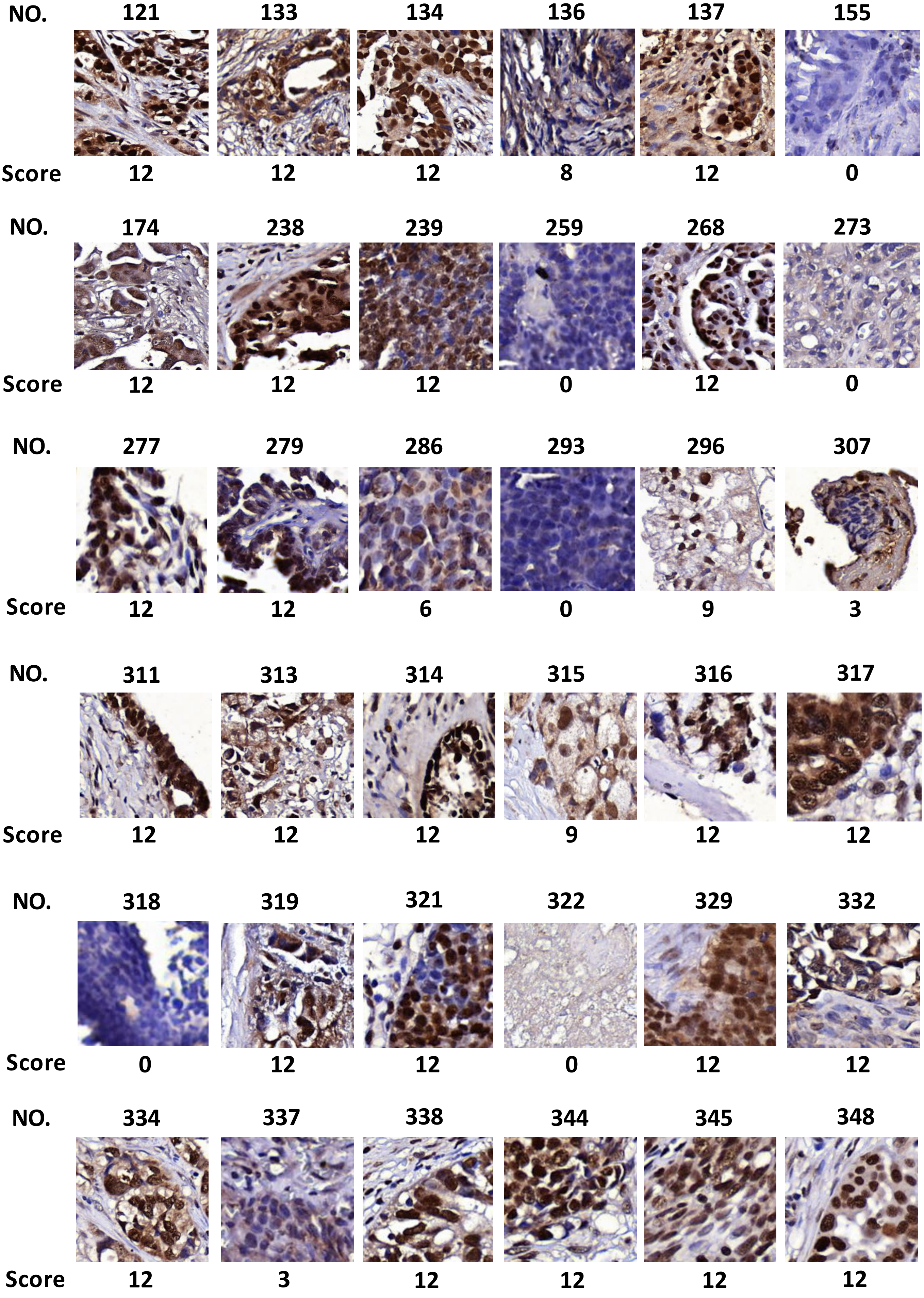

IHC of H3K27me3

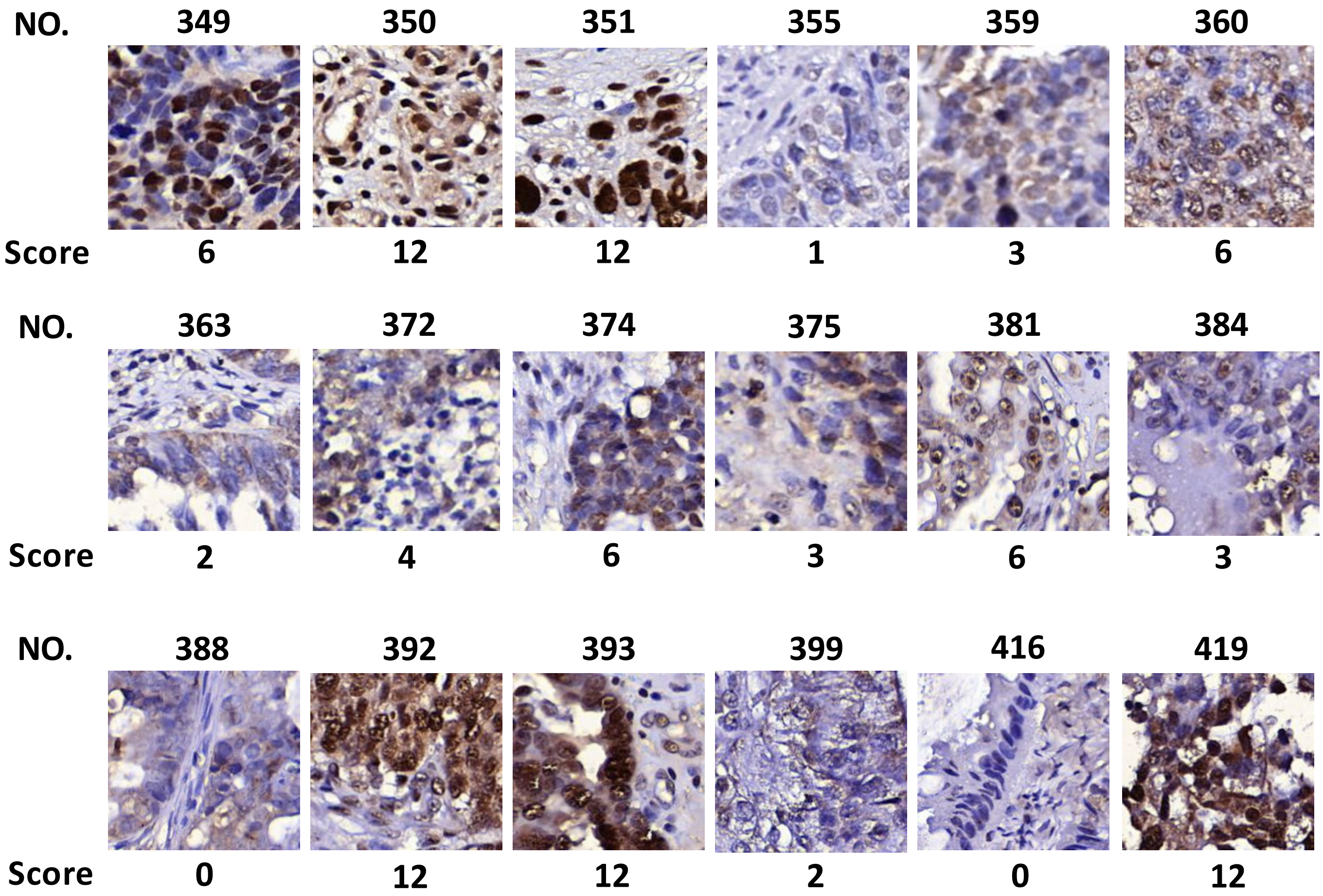

Supplement: File S1 [file peerj-08-9052-s001.pdf]
