# Supplementary figures and images for: EZH2/H3K27Me3 and phosphorylated EZH2 predict chemotherapy response and prognosis in ovarian cancer
Source: PeerJ. 2020 May 12;8:e9052. doi: 10.7717/peerj.9052 (PMC7227641; doi:10.7717/peerj.9052)

**IgG Negative**

**Positive**

**EZH2**

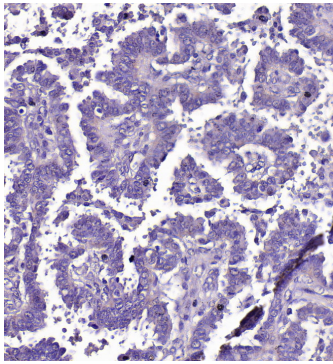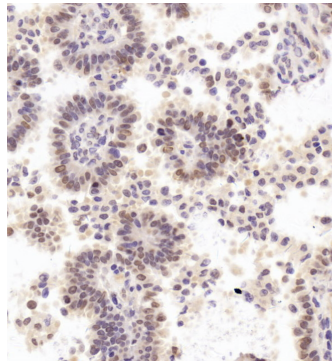

**p-EZH2**

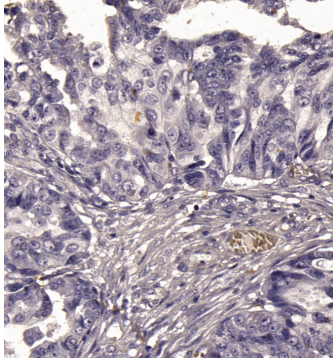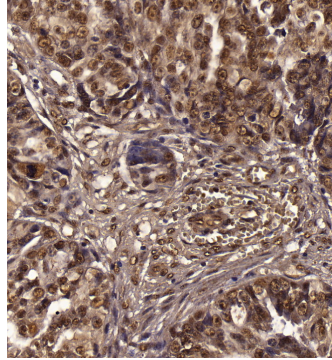

**H3K27me3**

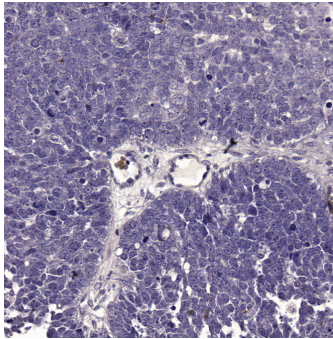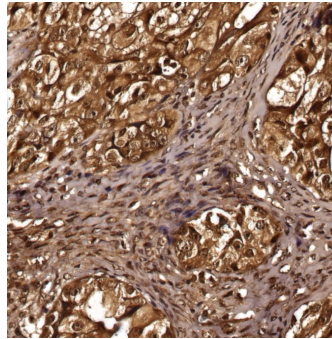

**p-Akt1**

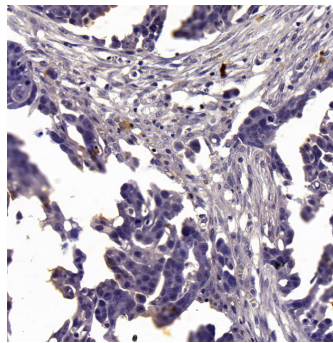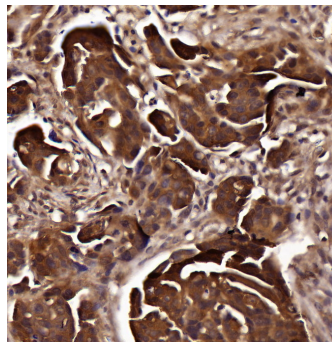

Supplement: File S3 [file peerj-08-9052-s003.pdf]
