# Supplementary material for: EZH2/H3K27Me3 and phosphorylated EZH2 predict chemotherapy response and prognosis in ovarian cancer
Source: PeerJ. 2020 May 12;8:e9052. doi: 10.7717/peerj.9052 (PMC7227641; doi:10.7717/peerj.9052)
Supplement: Supplemental Information 4 [file peerj-08-9052-s004.docx]

**Miame Checklist**

Experiment type: Parameter high vs low comparison

Parameters: EZH2, pEZH2 expression

Tissue type: human ovarian cancer tissues

Tissue microarray and IHC protocol:

All 65 non-consecutive, unselected primary ovarian cancer specimens were included in the tissue microarray. Formalin-fixed, paraffin-embedded (FFPE) tissue blocks were prepared according to the standard procedure. Tissue cylinders of 2 mm in diameter were punched from representative areas of each block with regard to the matching H&E staining control by a MiniCore Control Station (Alphelys Sarl, France). The Selected tissue cylinders were re-arranged and brought into three paraffin blocks by a semi-automated tissue arrayer (Beecher Instruments, Sun Prairie, WI, USA). 4 μm section slides were prepared for further use.

Immunohistochemistry (IHC) was performed as previously described. Briefly, the slides were dewaxed in xylene and went through a serial of descending ethanol to rehydrate. Antigen retrieval was performed through microwave irradiation. Blocking and staining were performed using Histostain Kits (SP9001 and SP9002, ZSGB-Bio, Beijing, China). Primary antibodies for EZH2 (1:100, Cell Signaling Technology, Danvers, USA), p-EZH2S21(1:100, Bethyal, Montgomery, USA), p-Akt1S473 (1:100, Cell Signaling Technology, Danvers, USA), EZH2 (1:100, Cell Signaling Technology, Danvers, USA) and H3K27Me3(1:400, Abclonal, Boston, USA) and were applied as recommended by the manufacturers. DAB color development and hematoxylin counterstaining were performed as appropriate.

Measurements：Each experimental images were quantified under the same exposure time using an Olympus, BX-51. The median IHC scores were set as the cut-off values of each experiment.
